# Upon the Digestibility of Iodide of Starch

**Published:** 1855-10

**Authors:** 


### *Upon the digestibility of Iodide of Starch.* By Dr. Jütte.

(Translated for the Examiner.)

Many physicians, when prescribing preparations of iodine, forbid the use of amylaceous food; acting upon the theory, that, from the great affinity of iodine to starch, the iodide of starch must be formed, which, as such, would pass from the body *undissolved*, whereby the action of the medicine would be weakened or wholly destroyed. The iodide of starch is, however, of so unstable a composition that it is easily decomposed, even by the saliva; the iodine entering again into soluble *absorbable* combinations, can be again recognised in the urine. In order to prove this, the author gave frequently the iodide of starch. It was prepared in the following manner: One ounce of wet starch was rubbed up with two drachms of tincture of iodine, and the mass dried. Of this powder there were taken, three times daily, ten grains, corresponding to one quarter of a grain of iodine at a dose. The examination of the urine was conducted in the following manner: The urine was mixed with some pulverized starch in a small proof glass, then a sufficient quantity of chlorine water was added; the previous white fluid became more or less violet-colored from the presence of the iodine; else a large quantity of the urine was evaporated to one tenth of its volume, then a few drops of sulphuric acid added, and immediately a paper spread with starch paste held over it. In every case when the iodide of starch had been taken, the author succeeded in detecting the presence of iodine in the urine. Sometimes it was difficult, immediately after the first dose of the iodide, to detect the iodine, there being but a slight reaction, on account of the insufficient sensibility of the reagent, which, however, becomes evident when several doses are given. In such cases, one can use the chloride of palladium, which is extremely sensitive, and leaves no doubt of the final passage of the iodine into the urine. It seems, therefore, unnecessary to deny the use of amylaceous food to patients while taking iodine.—*Günnsb. Zetschr.* v. 6. 1854., *Schmidt's Jahr.*

